# Comparing raccoon major histocompatibility complex diversity in native and introduced ranges: Evidence for the importance of functional immune diversity for adaptation and survival in novel environments

**DOI:** 10.1111/eva.12898

**Published:** 2019-11-28

**Authors:** Aleksandra Biedrzycka, Maciej Konopiński, Eric Hoffman, Alexa Trujillo, Andrzej Zalewski

**Affiliations:** ^1^ Institute of Nature Conservation Polish Academy of Sciences Kraków Poland; ^2^ Department of Biology University of Central Florida Orlando FL USA; ^3^ Mammal Research Institute Polish Academy of Sciences Białowieża Poland

**Keywords:** functional diversity, invasive species, major histocompatibility complex, *Procyon lotor*, raccoon, selection

## Abstract

The adaptive potential of invasive species is related to the genetic diversity of the invader, which is influenced by genetic drift and natural selection. Typically, the genetic diversity of invaders is studied with neutral genetic markers; however, the expectation of reduced diversity has not been consistently supported by empirical studies. Here, we describe and interpret genetic diversity at both neutral microsatellite loci and the immune‐related MHC‐DRB locus of native and invasive populations of raccoon to better understand of how drift and selection impact patterns of genetic diversity during the invasion process. We found that despite the loss of many MHC (major histocompatibility complex) alleles in comparison with native populations, functional MHC supertypes are preserved in the invasive region. In the native raccoon population, the number of supertypes within individuals was higher than expected under a neutral model. The high level of individual functional divergence may facilitate the adaptation to local conditions in the invasive range. In the invasive populations, we also detected increased population structure at microsatellites compared to the MHC locus, further suggesting that balancing selection is acting on adaptively important regions of the raccoon genome. Finally, we found that alleles known to exhibit resistance to rabies in the native range, *Prlo*‐DRB*4, *Prlo*‐DRB*16 and *Prlo*‐DRB*102, were the most common alleles in the European populations, suggesting directional selection is acting on this locus. Our research shows empirical support for the importance of functional immune diversity for adaptation and survival in novel environments.

## 1 INTRODUCTION

Understanding what makes some species able to become invasive is one of the central questions in conservation and evolutionary biology (Sakai et al., 2001). The ability of founders to adapt to a novel environment depends on genetic potential of the invader and habitat conditions of the novel environment (Willoughby, Harder, Tennessen, Scribner, & Christie, 2018). When the habitats are similar between native and introduced ranges, we might expect species to more readily become established. However, where environments differ, genetic diversity is important for the survival of the invaders (Reed & Frankham, 2003). The genetic diversity of introduced populations is related to the number of introduced individuals, the number of introduction events and the genetic characteristics of the native range (Kolbe et al., 2007; Roman & Darling, 2007; Zalewski et al., 2011). It has been argued that one of the most important conditions facilitating invasion success is high levels of genetic diversity due to the role of balancing selection in regard to adaptation to a novel environment (Lee, 2016). Biological invasions and subsequent range expansions create asymmetries between the individuals that are invaders and individuals living in the native range. These differences mainly arise due to founder events which may lead to reduced genetic diversity in the invasive range (Blackburn, Lockwood, & Cassey, 2015; Tsutsui, Suarez, Holway, & Case, 2000). As a consequence of the foundation process, introduced populations are often characterized by lower genetic diversity compared to source populations (Monzón‐Argüello, Leaniz, Gajardo, & Consuegra, 2014). On the other hand, relatively high levels of genetic variation may be brought to the invasive range by individuals originating from diverse source populations, as well as multiple waves of invasion from different native populations that hybridize in the new range. This increased allelic diversity could lead to novel gene combinations in the invasive range that may provide resistance to pathogenic infections (Sakai et al., 2001). If the invasion of non‐native species consists of numerous dispersal events and high gene flow between areas (Kolbe et al., 2007; Zalewski, Michalska‐Parda, Bartoszewicz, Kozakiewicz, & Brzeziński, 2010), invasive populations should exhibit minimal genetic structuring throughout the introduced range (Zalewski et al., 2010). Over time, during the expansion process of a successful invader, genetic variation becomes partitioned among populations via genetic drift and adaptation to local conditions. There is broad evidence to support the fact that although successful invaders frequently experience genetic bottlenecks (Tsutsui et al., 2000), the effects are not severe, due to relatively large and diverse (Posavi, Gelembiuk, Larget, & Lee, 2014) founder populations and multiple introduction events (Kolbe et al., 2007). Therefore, the genetic variation within invasive populations is often retained at levels similar to native populations (Dlugosch, Anderson, Braasch, Cang, & Gillette, 2015).

Although genetic variation of invaders has been extensively explored over the past decades, most of the studies utilize total genetic diversity or focus on neutral genetic variation that serves as a proxy for adaptive genetic variation (Uller & Leimu, 2011; Wellband, Pettitt‐Wade, Fisk, & Heath, 2018). Studying genetic diversity and population structure of adaptively important genes in invasive species is crucial for recognizing the impacts of microevolutionary changes on invasion success (reviewed by Dlugosch et al., 2015; White & Perkins, 2012) and provides a theoretical background for the management of populations of invasive species (Kelly, Paterson, Townsend, Poulin, & Tompkins, 2009; Torchin, Lafferty, Dobson, McKenzie, & Kuris, 2003). Namely, in immune‐related genes, we expect weaker pathogen pressure at the invasion front resulting in relaxed selection, while previously established core populations may carry a higher diversity of pathogens and hence experience stronger balancing selection, leading to a higher immune genetic diversity (White & Perkins, 2012).

Genes that compose the major histocompatibility complex (MHC) capture an important fraction of the genetic variation underpinning resistance to pathogens (Acevedo‐Whitehouse & Cunningham, 2006) and have proved to be useful in examining the adaptive potential of populations in mammalian species (Aguilar et al., 2004; de Assunção‐Franco, Hoffman, Harwood, & Amos, 2012; Siddle, Marzec, Cheng, Jones, & Belov, 2010). Under balancing selection, differentiation between populations has been predicted to be significantly reduced compared with neutral loci, while under positive selection, we should expect greater differentiation at functional immune loci than at neutral loci due to different selective agents acting in different populations (Bernatchez & Landry, 2003). One of the forms of balancing selection is “*divergent allele advantage*” (Lenz, 2011) where if the two alleles in a heterozygous genotype are highly divergent, they should provide more comprehensive immune surveillance than genotypes with less divergent alleles. In the case of MHC genes, selection may act on functional MHC variants represented by MHC supertypes rather than on specific alleles.

The loss of MHC diversity in invasive species is thought to be due to a combination of drift, relaxed selection and directional selection. However, the way this loss occurs at different stages of invasion is largely unknown. In practice, a number of empirical studies showed that selection acting on immune diversity was not strong enough to counteract the loss of diversity induced by genetic drift (Alcaide, 2010; Grueber, Wallis, & Jamieson, 2013; Miller, Allendorf, & Daugherty, 2010; Sutton, Nakagawa, Robertson, & Jamieson, 2011). Other studies reported that balancing selection may also prevent the loss of immunogenetic diversity induced by strong bottlenecks (Monzón‐Argüello et al., 2014; Oliver & Piertney, 2012; Strand et al., 2012).

The raccoon, *Procyon lotor*, is a medium‐sized carnivore whose native distribution in North America extends from southern Canada to Panama (Zevelhof, 2002). Raccoons were successfully introduced in Germany, in the 1930s with a limited number of individuals (Jernelöv, 2017). Recent genetic analyses suggest that there were at least four small‐scale, independent initial introduction events of raccoon (Fischer et al., 2017). Recently, approximately 1,000,000 raccoons were estimated to be living in Germany, and the range of the species in Europe has extended to the West, East and South of the invasion core (Bartoszewicz, Okarma, Zalewski, & Szczesna, 2008; Lutz, 1984). A recent study used neutral genetic markers and found genetic structuring throughout the European range (Biedrzycka, Zalewski, Bartoszewicz, Okarma, & Jędrzejewska, 2014).

In this study, we aimed to characterize and interpret genetic variation and differentiation in invasive raccoons at the immune locus MHC‐DRB to determine if and to what extent functional genetic diversity is maintained in highly successful invasive species. First, to test for positive selection acting on the MHC‐DRB locus, we performed site‐specific tests of selection. Given that the main function of MHC molecules is to present a wide range of antigens to the immune system, we expected to find positive selection acting on specific sites within a coding sequence (Hughes & Nei, 1988; Kosakovsky Pond & Frost, 2005). Second, we described MHC‐DRB population and individual diversity from the native raccoon population, the invasion core and several sites across the invasion gradient. To evaluate whether the amount of individual functional variation retained in invasive populations is affected by selection, we compared the number of mean individual immune supertypes in native and invasive ranges with the data obtained under a neutral model. We expected to find lower levels of genetic diversity in the invasive populations compared to the native populations and changes in genetic diversity along the invasion gradient. Lastly, we compared MHC‐DRB and microsatellite interpopulation differentiation to account for demographic processes in the invasive range. We expected that genetic drift and relaxed selection pressure during the colonization process will similarly impact neutral and immune‐related genes. Alternatively, pathogen‐mediated selection acting on the MHC‐DRB locus may have maintained population‐specific polymorphism due to spatial variation in pathogen exposure or may equalize allele frequencies as a result of balancing selection.

## 2 MATERIALS AND METHODS

### 2.1 Study area and sampling methods

**Figure 1:**
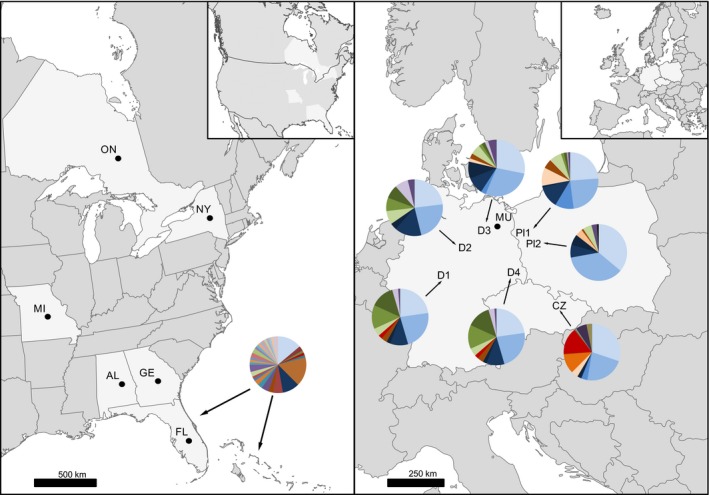
Geographic locations of native (left panel) and invasive (right panel) raccoon populations. MHC‐DRB allele frequencies shown by the pie charts. The numbers correspond to the ones used in Table 1 and Figure 2. The sampling sites are located in Hesse (D1), Saxony‐Anhalt (D2), Mecklenburg (D3), Saxony (D4), Warta Mouth National Park (PL1), Warta Mouth National Park surroundings (PL2) and Czech Republic (CZ). The dots represent locations where previously published alleles were sampled

We collected DNA samples from the native (USA) and introduced (Europe) range of raccoons. Raccoons from the native range (*n* = 21) were sampled from six different localities in mainland Florida (USA) and surrounding islands as representatives of different subspecies. These samples were obtained from local pest control programmes (Trujillo & Hoffman, 2017). From the introduced range, we collected 317 samples between 2012 and 2017 (Table 1). Muscle tissue samples were obtained from hunters culling raccoons as part of game management activity in Poland, Germany and Czechia. The geographic locations of the samples are presented in Figure 1. All tissue samples were stored at −20°C prior to DNA extraction. For all collected samples, we obtained microsatellite and MHC genotypes. Additionally, we used all available sequences for raccoon exon 2 MHC‐DRB locus deposited in GenBank. This comprised alleles discovered by Castillo, Srithayakumar, Meunier, and Kyle (2010) and Ruiz‐López et al. (2014) from the native range of raccoon and sequences from Santos, Michler, and Sommer (2017) detected in the invasive range, in Germany. This enabled us to get insight to raccoon MHC diversity from the possibly widest range in both native and invasive populations. Because of slight differences in sequence length between sequences obtained in different studies, we were not able to distinguish between some unique alleles identified in other studies. The localities from which alleles were sampled are presented in Figure 1, and the list of all alleles used along with references is presented in Table S1.

**Table 1:** Species‐wide diversity of MHC‐DRB locus

Site	*n*	*A*	*A*_priv_	*N*_s_	*A*_R_	IndA_mean_ (*SD*)	IndS_mean_	*S*	*π*	*π*_syn_	*π*_nonsyn_	Ind_p‐distance
**Native** (GenBank alleles)	**–**	76	**–**	10	**–**	**–**	**–**	51	0.079	0.057	0.086	–
**Invasive** (GenBank alleles)	**–**	28	**–**	9	**–**	**–**	**–**	46	0.089	0.073	0.093	**–**
**Native** (sampled individuals)	21	32	26	9	22.06	3.43 (0.660)	2.81	49	0.090	0.064	0.098	0.603
**Invasive** (sampled individuals)	317	20	14	9	8.83	3.13 (1.006)		48	0.080	0.075	0.101	
D1	16	9	0	6	7.20	3.50 (1.323)	2.63	45	0.0928	0.0870	0.0946	0.712
D2	7	12	0	5	9.00	2.97 (0.897)	2.86	46	0.0722	0.0555	0.0776	0.705
D3	35	9	0	6	7.84	3.71 (0.670)	2.43	33	0.0726	0.0486	0.0802	0.782
D4	27	12	0	5	7.89	3.36 (1.008)	2.79	44	0.0746	0.0527	0.0815	0.722
PL1	107	14	0	5	7.77	3.42 (1.098)	2.59	47	0.0886	0.0748	0.0931	0.777
PL2	36	9	0	6	5.72	2.62 (0.967)	2.25	40	0.0604	0.0424	0.0661	0.815
CZ	89	14	2	7	7.12	3.20 (0.876)	2.33	46	0.0805	0.065	0.085	0.697

Abbreviations: *A*, number of alleles; *A*_priv_, number of private alleles; *A*_R_, allelic richness; Ind_p‐distance, mean individual amino acid distance for positively selected sites; IndA_mean_ (*SD*), mean number of alleles per individual; IndS_mean_, mean number of supertypes per individual; *k*_a_, average number of nonsynonymous nucleotide differences between alleles; *n*, number of individuals; *N*_S_, number of supertypes; *S*, number of segregating sites; *π*, nucleotide diversity per site.

### 2.2 DNA isolation, microsatellite genotyping, MHC‐DRB locus amplification and genotyping

For samples collected from the native range, DNA was extracted using Qiagen DNeasy Blood and Tissue Kit or a Serapure Bead (Rohland & Reich, 2012) extraction method. For samples collected in Europe, DNA was extracted from dried ear fragments or ethanol‐preserved tissue using the NucleoSpin Tissue Kit (Macherey and Nagel) according to the manufacturer's protocol. Individual samples were genotyped at nine microsatellite loci (Cullingham, Kyle, & White, 2006; Fike, Schreier, Beasley, Dharmarajan, & Rhodes, 2007) according to the protocol described in Biedrzycka et al. (2014). Amplification products were genotyped on an ABI 3130xl Genetic Analyzer using GeneMapper software v. 5.0 (Applied Biosystems).

To analyse the diversity of the MHC‐DRB locus, we amplified a 184‐bp (excluding primers) fragment of exon 2 that contains the functionally important protein binding region (PBR), using DRB 5c and DRB 3c primers (forward: 5′ TCAATGGGACGGAGCGGGTGC 3′, reverse: 5′ CCGCTGCACAGTGAAACTCTC 3′) previously used for MHC characterization in a native raccoon population by Castillo et al. (2010). The sequencing primers consisted of DRB 5c or DRB 3c primers followed by a unique 6‐bp barcode and Illumina‐specific primers. Amplification was performed with HotStar Master Mix (QIAGEN), and the reaction was run for 27 cycles at 95°C for 30 s, 66°C for 30 s and 72°C for 1 min 30 s. We used a combination of 12 forward and eight reverse uniquely barcoded primers in 96‐well PCR plates. The amplicons in each row shared the same barcode at the forward primer and differed in the barcode at the reverse primer, which resulted in a unique tag combination in each well. We included one negative control per 16 samples, and 24 samples were run as duplicates to control for sequencing errors.

The concentration of PCR products was estimated by eye from agarose gels, and products from each row were pooled into approximately equimolar quantities. The pools were then purified using MinElute PCR Purification Kit (QIAGEN). Next, each pool was diluted between 10 and 100×, depending on the electrophoresis band intensity. The pooled amplicons were amplified in multiplexed PCR with primers including Illumina P5/P7 sequences, resulting in Illumina‐compatible paired‐end sequencing templates (Syed, Grunenwald, & Caruccio, 2009). The products obtained were again run on the agarose gel to visually assess product intensity and were purified with the MinElute PCR Purification Kit (QIAGEN). Paired‐end sequencing runs were performed on an Illumina MiSeq machine with MiSeq Reagent Kit v2 for 300 cycles (Illumina, Inc.). The samples were genotyped in four independent runs as we had only 96 different barcode combinations. The read merging, filtering, quality control and preliminary control of length, coverage and frequency of the most abundant variants, as well as final genotyping of MHC‐DRB locus, were performed using the AmpliSAS pipeline (Sebastian, Herdegen, Migalska, & Radwan, 2016). To preliminary explore the data set, the first run was analysed with AmpliCHECK using default Illumina parameters. For final genotyping, we adopted a minimum per‐amplicon frequency threshold of 2%. A maximum of 5,000 reads per amplicon were used for genotyping to reduce computational load.

### 2.3 MHC‐DRB phylogenetic relationships and tests of historical selection

The unique sequences were aligned, edited and interpreted using BioEdit (Hall, 1999). To describe phylogenetic relationships among MHC‐DRB alleles, that is to check for allele clustering patterns that would imply trans‐population evolution (Klein, Sato, & Nikolaidis, 2007), we created a neighbour‐net network. The evidence for clustering of alleles would also enable approximate assignment of alleles into separate loci. The network was also intended to show possible reticular relationships originating from gene duplication and recombination in MHC‐DRB locus. The neighbour‐net network was constructed in SplitsTree v.4 (Huson & Bryant, 2006) with edge weights estimated using ordinary least squares variance and a threshold of 10. The network was built under a JC+G model of nucleotide substitution as identified in MEGA 7 (Kumar, Stecher, & Tamura, 2016) with branch support assessed via bootstrap (1,000 replicates).

To check if the signatures of positive selection at the molecular level, typically found in MHC genes (Bernatchez & Landry, 2003; Hughes & Nei, 1988), affected raccoon MHC‐DRB gene diversity, we tested for evidence of selection at specific codons using phylogenetically controlled selection tests based on d*N*/d*S* ratios (Kosakovsky Pond & Frost, 2005). All MHC alleles were used in the selection tests without differentiating native and invasive alleles as we aimed to detect historic, not contemporary, selection. The analyses were performed using the program HyPhy implemented on the Datamonkey server (Delport, Poon, Frost, & Kosakovsky Pond, 2010). First, we tested for possible recombination events that might have affected the diversity of each locus using Genetic Algorithm Recombination Detection (GARD; Pond, Posada, Gravenor, Woelk, & Frost, 2006), as recombination events can affect the outcome of selection tests. We then ran a model selection procedure to identify the model that best fit the data to use for the following steps. Next, we ran phylogenetically controlled models of selection: MEME, which allows for detecting episodic positive selection (Murrell et al., 2012); FUBAR, which allows for the detection of sites under positive or purifying selection (Murrell et al., 2013); SLAC, which infers sites under positive and negative selection; and FEL, which uses maximum‐likelihood approach to identify sites under positive and selection assuming constant selection pressure (Kosakovsky Pond & Frost, 2005). As the results of these tests could differ from one another, to follow conservative approach, we only considered a site to be under selection if this was indicated by at least three out of four tests. The maximum‐likelihood trees were then used to obtain branch lengths and substitution rates (Delport et al., 2010).

### 2.4 Supertype clustering

To investigate the significance of functional MHC class I diversity, we clustered alleles into supertypes. MHC alleles of the same supertype encode biochemically similar amino acids at antigen‐binding sites, and thus, the molecules bind similar antigens, whereas molecules encoded by alleles from different supertypes recognize repertoires of different antigens. Therefore, alleles of different supertypes should have different functional values (Doytchinova & Flower, 2005; Trachtenberg et al., 2003). Clustering was based on the physicochemical properties of positively selected amino acid sites (PSS), that is sites with a high rate of nonsynonymous substitution indicative of an important role in antigen binding specificity (Hughes & Nei, 1988). Each PSS was substituted by a set of five physicochemical descriptors (Sandberg, Eriksson, Jonsson, Sjöström, & Wold, 1998); then, we used the R package “adegenet” (Jombart, Devillard, & Balloux, 2010) to perform k‐means clustering and discriminant function of principal components (DAPC). The number of clusters was then chosen based on the graph of BIC (Bayesian information criterion) values for increasing number of clusters. The most probable number of supertypes in our data set was defined as the minimal number of clusters after which the BIC decreases by a negligible amount (Jombart et al., 2010). The number of principal components (PCs) retained in DAPC was chosen to maximize the α‐score (using the *optim.a.score* function of adegenet).

### 2.5 MHC‐DRB allelic diversity and supertype abundance

To assess the levels MHC‐DRB diversity in raccoon populations from different localities, we used DnaSP v.5 (Rozas, 2009). P‐distances of nucleotides and amino acid sequences were estimated using MEGA7 (Kumar et al., 2016). We calculated number of alleles per population (*A*), mean number of alleles per individual, the number of private alleles (*A*_priv_), the number of segregating sites (*S*) and nucleotide diversity (*π*) for whole sequences as well as for synonymous and nonsynonymous sites separately. We used SPAGeDi (Hardy & Vekemans, 2002) to estimate per population allelic richness (*A*_R_), expressed as the expected number of alleles assuming the smallest sample size (i.e. seven individuals). To evaluate whether the individual number of functional MHC‐DRB variants (represented by MHC supertypes) in native and invasive populations differs from that expected under neutrality, we compared the actual distribution of supertypes observed in individuals to a null distribution obtained from randomly generated genotypes. Because different MHC alleles may be linked within individual genotypes either by disassortative mating or due to spatial proximity of different alleles within the genome (i.e. physical linkage), we tested for possible associations between alleles using *pair.ia* function from *poppr* R package (Kamvar, Tabima, & Grünwald, 2014). The function was used to estimate indexes of association *I*_A_ (Smith, Smith, O'Rourke, & Spratt, 1993) between each pair of alleles and their p‐value derived from 99,999 permutations. A Bonferroni correction was used to account for multiple comparisons. Artificial genotypes were simulated assuming associations between alleles according to their pairwise *I*_A_ and entirely random occurrence. Each simulated set consisted of 50,000 genotypes. We used a Kolmogorov–Smirnov test to evaluate whether differences occurred between the actual distribution of supertypes compared to a null distribution of supertypes. To account for the discrete character of the data, we used *ks.test* function from *dgof* R package (Arnold & Emerson, 2011), with the null distribution transformed using *ecdf* routine. All simulations and statistical tests were performed in R (R core Team, 2014), and the script has been deposited in the Dryad database.

### 2.6 Population structure at MHC and microsatellite loci

To address our question of the relative roles of demographic and selective forces in shaping MHC‐DRB diversity, we assessed population structure at MHC and microsatellite loci. Initially, we estimated MHC allele frequencies at specific sample sites (Figure 1). In multilocus genes, such as MHC, alleles cannot usually be assigned to loci, due to gene conversion, allele homogenization among loci (Klein, Satta, O'hUigin, & Takahata, 1993), and other processes that create closely linked duplicated MHC loci and copy number variation among and within species (Mehta, Nonaka, & Nonaka, 2009). Difficulty in assigning alleles to loci makes the identification of heterozygote and homozygote genotypes and the estimation of allele frequencies difficult or impossible. To analyse among‐site genetic differentiation at the MHC‐DRB locus, we used the metric Rho (Ronfort, Jenczewski, Bataillon, & Rousset, 1998)*. *Rho is independent of both the ploidy level and the amount of within‐individual diversity, making it an ideal statistic for multilocus genes such as MHC, where it is not possible to assign specific alleles to loci and where there may be violations of the assumptions of Hardy–Weinberg equilibrium, linkage disequilibrium and mixed inheritance patterns. The level of interpopulation differentiation at neutral markers was calculated as pairwise Rho for microsatellites. To account for nonlinear population distribution and migration patterns (Rousset, 1997), pairwise Rho values were transformed to Rho/(1 − Rho). The analyses were performed using SPAGeDi (Hardy & Vekemans, 2002). To compare the levels of substructuring displayed by two different types of markers, we performed Mantel test and partial Mantel (Pearson statistics) test using Vegan v. 2.5‐4 package in R (Oksanen et al., 2016). To assess patterns of isolation by distance, we first tested for a relationship between microsatellite loci and geographic distance and for a relationship between MHC‐DRB locus and geographic distance, independently. Second, isolation by distance at the MHC‐DRB locus was further tested comparing Rho/(1 − Rho) for MHC and geographic distance while controlling for neutral pairwise differentiation (Rho/(1 − Rho) for microsatellites). Finally, we evaluated the correlation between pairwise estimates of Rho/(1 − Rho) for microsatellites and MHC‐DRB while controlling for geographic distance. The significance of all correlations was assessed with 719 matrix permutations.

To further test for population structure displayed at the MHC‐DRB locus and at microsatellite loci, we used discriminant analysis of principal components (DAPC) implemented in the R package adegenet (Jombart et al., 2010). Multivariate analyses are the method of choice when the assumptions of Hardy–Weinberg and linkage equilibrium within populations are violated. DAPC uses principal component analysis (PCA) to reduce the number of variables used in discriminant analysis (DA) to only use the variables that are uncorrelated. This approach maximizes between‐group differences while minimizing within‐group variance, thus enabling DAPC to detect subtle differences between populations. First, using the function *find.clusters*, we identified the number of clusters that best reflects the genetic structuring in the data without a prior assignment of samples to given populations, using BIC scores (Bayesian information criterion; Jombart et al., 2010). A cross‐validation function *(Xval*.*Dapc*) was used to select the optimal number of principal components to be retained. Next, we used DAPC to generate assignment probabilities to actual populations (sampled sites). Due to the fact that we detected hierarchically arranged levels of genetic structure in invasive raccoon populations in our previous study (Biedrzycka et al., 2014), we tested genetic structure using the same multiple levels in the present analysis. For microsatellite data, we ran three different iterations to determine the pattern of genetic structuring best supported by the data. First, we included all samples from both native and invasive populations. Second, we investigated substructuring across different levels of the landscape. Here, we (a) excluded all samples from the native range; then, we (b) excluded the population from Czechia, as it formed a clear separate cluster. For MHC‐DRB data, we used the same levels of hierarchical structuring, omitting the native raccoon population because the different allelic composition of native population compared to invasive populations.

## 3 RESULTS

### 3.1 Microsatellite genotyping, MHC‐DRB locus amplification and genotyping

For microsatellite genotyping, the list of loci used, along with their diversity indices, is presented in Table S2. With regard to MHC‐DRB amplification, we sequenced a 184‐bp fragment of the MHC‐DRB locus, exon 2, in 338 racoons (317 from invasive range and 21 from native range). The individual amplicon coverage depth ranged from 334 to 7,970 reads per individual. The AmpliSAS pipeline revealed only one sequence length with no insertions or deletions, and we also did not detect any stop codons or other signs suggesting presence of pseudogenes. We found from 2 to 6 variants per individual suggesting copy number variation in raccoon MHC‐DRB locus. We detected a high number of variants that appeared only once among sampled individuals, especially in native range. Taking into account extremely high level of MHC diversity and limited number of samples from the native range, we decided to keep these variants providing that their frequencies in a given amplicon were higher than 10% and were previously detected in other studies. We obtained 100% repeatability between 24 replicates applying the above criteria. As in most species, it was not possible to assign detected alleles to specific MHC‐DRB loci (Eirín‐López, Rebordinos, Rooney, & Rozas, 2012; Miller & Lambert, 2004); however, for simplicity, we decided to call all identified variants “alleles” although, strictly speaking, this is a collection of alleles at many loci, and used the total individual number of “alleles” as the measure that reflects individual MHC‐DRB diversity across all present loci. We also refer to MHC‐DRB locus as we are not able to verify the exact number of multiple loci present in each individual.

### 3.2 MHC‐DRB phylogenetic relationships, tests of historical selection, and supertype diversity

We identified 43 putatively expressed alleles, among individuals from native and invasive populations (Table S1); 37 alleles were previously described in either native raccoon populations (Castillo et al., 2010) or invasive populations (Santos et al., 2017). Seven novel alleles were detected in this study and submitted to GenBank with the accession numbers MK028988–MK028994 and were assigned new designations, from Prlo‐DRB*264 to Prlo‐DRB*270 (the numbers of the alleles were ordered sequentially according to abundance in our sample). Two of those alleles (Prlo‐DRB*270 and Prlo‐DRB*271) were found in invasive populations, and the remaining alleles were found in native populations from Florida. There was no redundancy among MHC‐DRB alleles detected in our study; that is, every allele translated into a unique amino acid sequence. When previously detected alleles, drawn from the GenBank, were added to our data set, we analysed 89 unique sequences (76 form the native range and 28 from the invasive range). The GARD test of recombination did not find any recombination events between tested alleles. The test of site‐specific selection found five sites that were consistent between all four tests, the additional sites were detected by less than three tests, and we did not consider them as positively selected, according to adopted approach.

The clustering procedure revealed 10 different supertypes, and the number of alleles assigned to each supertype ranged from 2 to 16 (Table S1). The number of alleles per individual ranged from two to four in the native range and from two to six in the invasive population, suggesting the presence of at least three MHC‐DRB loci. The number of supertypes per individual ranged from two to three and from one to four for native and invasive populations, respectively. The number of alleles and supertypes per individual was strongly correlated for both native and invasive populations (0.683 and 0.775, respectively). The mean number of alleles/supertypes per individual was 3.13/2.51 for invasive and 3.43/2.81 for native populations. We detected 20 different MHC‐DRB alleles among 317 raccoon samples from European invasive populations and 32 alleles in 21 samples from native raccoon population from Florida (Table 1).

**Figure 2:**
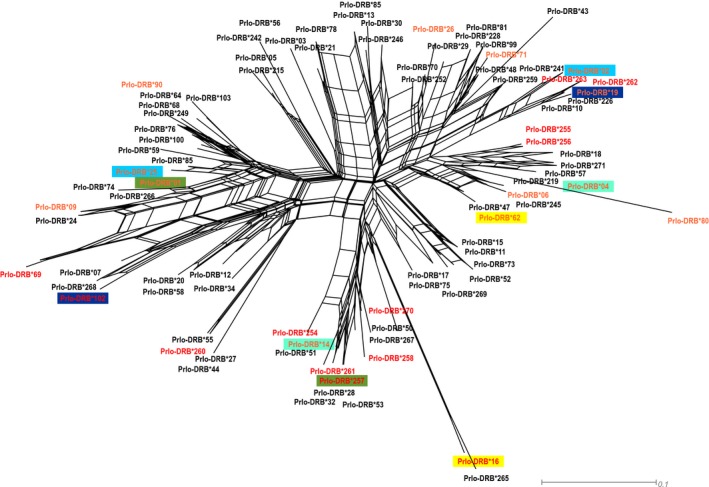
Neighbour‐net network of raccoon MHC‐DRB alleles constructed from alleles detected in this study and previously reported in the literature. The loops imply areas of phylogenetic uncertainty or reticulations. The alleles marked with red were detected only in invasive range, and orange alleles were detected in both native and invasive ranges. Alleles marked with black were detected only in the native range. Allele pairs marked with different colours were tightly associated in invasive population

The allele network constructed for all 89 alleles did not reveal any clear and significant patterns of clustering that would suggest grouping alleles form different loci and showed high level of similarity between alleles belonging to different loci in raccoon (Figure 2). The reticular connections between alleles are suggestive of gene duplication followed by recombination and gene conversion in MHC‐DRB locus. Such phylogenetic intermingling of alleles from different loci is indicative of homogenizing concerted evolution. The bootstrap values separating alleles in the network core were low and did not exceed 50% in any case (Figure 2). We also did not detect any signs of allele clustering between native and invasive ranges as alleles from the native and invasive ranges were spread throughout the network. This result is in line with trans‐population evolution of MHC‐DRB locus.

For MHC‐DRB diversity, the native population exhibited the highest number of alleles and allelic diversity, but the estimates of average nucleotide diversity per site (*π*) in synonymous and nonsynonymous sites were similar for native and invasive populations. Clustering analyses revealed 10 unique supertypes among all available MHC‐DRB alleles and nine supertypes among individuals sampled in the native range (Table 1). Among different European sampling sites, the number of alleles per site ranged from 9 in D1, D3 and PL2 to 14 in CZ and PL1. The number of private alleles was low, with only two in CZ and no private alleles in the remaining sites (Table 1). The allelic richness (*A*_R_) ranged from the highest at the D2 sampling site (9.00) to the lowest in PL2 (5.72). The raccoons from D1, considered to be the site of the initial raccoon introduction, displayed the highest estimates of both synonymous and nonsynonymous nucleotide diversity per site, despite a low number of alleles. Within different European locations, the number of supertypes ranged from 5 to 7 (Table 1).

A comparison of the number of supertypes in observed and simulated data sets was performed on three separate raccoon groups. We treated separately (a) individuals from native population (Florida); (b) individuals from Czechia (site CZ), because genetic structure at the MHC‐DRB locus (DAPC clustering; see below) and numerous lines of evidence (Biedrzycka et al., 2014) indicate that this population originated from a separate introduction event and presents different allelic patterns; and (c) individuals from Poland and Germany (sites PL and D), because we did not detect any visible population structure between them (DAPC clustering; see below). Allele association analyses indicated nonrandom segregation of certain allele pairs in the invasive raccoon population. Significant association was detected between one allele pair in CZ: Prlo‐DRB*16/Prlo‐DRB*62 (*I*_A_ = 1.00, *p* < 10^–5^), while five pairs showed significant association within the PL and D populations: Prlo‐DRB*04/Prlo‐DRB*14 (*I*_A_ = 0.93, *p* < 10^–5^), Prlo‐DRB*16/Prlo‐DRB*62 (*I*_A_ = 0.76, *p* < 10^–5^), Prlo‐DRB*19/Prlo‐DRB*102 (*I*_A_ = 0.5, *p* < 10^–5^), Prlo‐DRB*02/Prlo‐DRB*25 (*I*_A_ = 0.71, *p* < 10^–5^) and Prlo‐DRB*01/Prlo‐DRB*257 (*I*_A_ = 1.00, *p* < 10^–5^). Although no significant associations between alleles were found in population sample from Florida, we are aware that the lack of associations between alleles can be a result of much smaller sample size.

Comparisons of the number of supertypes between observed and simulated data sets showed that in German and Polish populations, the observed mean number of supertypes is higher than simulated without assuming association between alleles (2.56 and 2.47, respectively, *p* = .003), but lower than simulated when complete association is assumed (2.71, *p* = .0001). No significant differences were observed in the CZ population (observed—2.33; unlinked—2.32; fully linked—2.33). Because no associations were found in the FL population, the only possible comparison was between observed (2.81) and simulated unlinked alleles (2.00). Here, the difference was statistically significant (*p* = 3 × 10^–7^).

### 3.3 Population structure at MHC and microsatellite loci

To evaluate population structure, we first estimated MHC‐DRB allele frequencies among populations. Among the 20 different alleles present in the European raccoon population, alleles Prlo‐DRB*04 and Prlo‐DRB*14 were typically present in high frequency in all sites (frequencies ranged from 0.76 to 0.97 and 0.71 to 0.97, respectively). The only exception was D1 where, despite relatively low sample size, allele frequencies were evenly distributed, with no pronounced dominance of Prlo‐DRB*04 and Prlo‐DRB*14 alleles, and only one allele exhibited a frequency lower than 10% (Figure 1). The number of alleles with a frequency lower than 10% ranged from 3 to 6 among the remaining introduced sites.

Global among‐site differentiation expressed as Rho/(1 − Rho) was 0.190 and 0.240 for MHC‐DRB and microsatellite loci, respectively. Despite the absence of private alleles among European sites (but CZ; see above), the MHC‐DRB population structure was high and significant for most comparisons. However, no differentiation was found between D2 and D3, and PL1 sites (Table 2). The pairwise differentiation at microsatellite loci was significant for all pairwise comparisons, including sites for which the differentiation was nonsignificant at the MHC‐DRB locus (Table 2). The pattern of isolation by distance was significant for microsatellites (*r* = .513, *p* = .03), but not for the MHC‐DRB locus (*r* = .429, *p* = .098). When the correlation between MHC‐DRB and geographic pairwise distances was controlled for neutral diversity, the pattern was also not significant (*r* = .391, *p* = .132). We also did not find an association between MHC‐DRB and microsatellite interpopulation diversity while controlling for geographic distance (*r* = −.038, *p* = .569).

**Table 2:** Pairwise Rho calculated for MHC locus (above) and microsatellites (below) expressed as Rho/(1 − Rho)

MHC	D1	D2	D3	D4	PL1	PL2	CZ
D1	0	0.253	0.386	0.417	0.248	1.016	0.518
D2	**	0	0.056	−0.024	0.040	0.469	0.270
D3	***	NS	0	0.141	0.092	0.064	0.177
D4	***	NS	**	0	0.143	0.419	0.272
PL1	***	NS	***	***	0	0.265	0.233
PL2	***	**	**	***	***	0	0.281
CZ	***	***	***	***	***	***	0
**Microsatellites**	**D1**	**D2**	**D3**	**D4**	**PL1**	**PL2**	**CZ**
D1	0	0.219	0.099	0.183	0.420	0.340	0.455
D2	***	0	0.138	0.212	0.270	0.294	0.378
D3	*	**	0	0.083	0.313	0.246	0.334
D4	***	***	*	0	0.112	0.068	0.352
PL1	***	***	***	***	0	0.077	0.480
PL2	***	***	***	***	***	0	0.513
CZ	***	***	***	***	***	***	0

Note

The significance level is given below the diagonal.

Abbreviation: NS, not significant.

* *p* < .05;

** *p* < .01;

*** *p* < .001.

**Figure 3:**
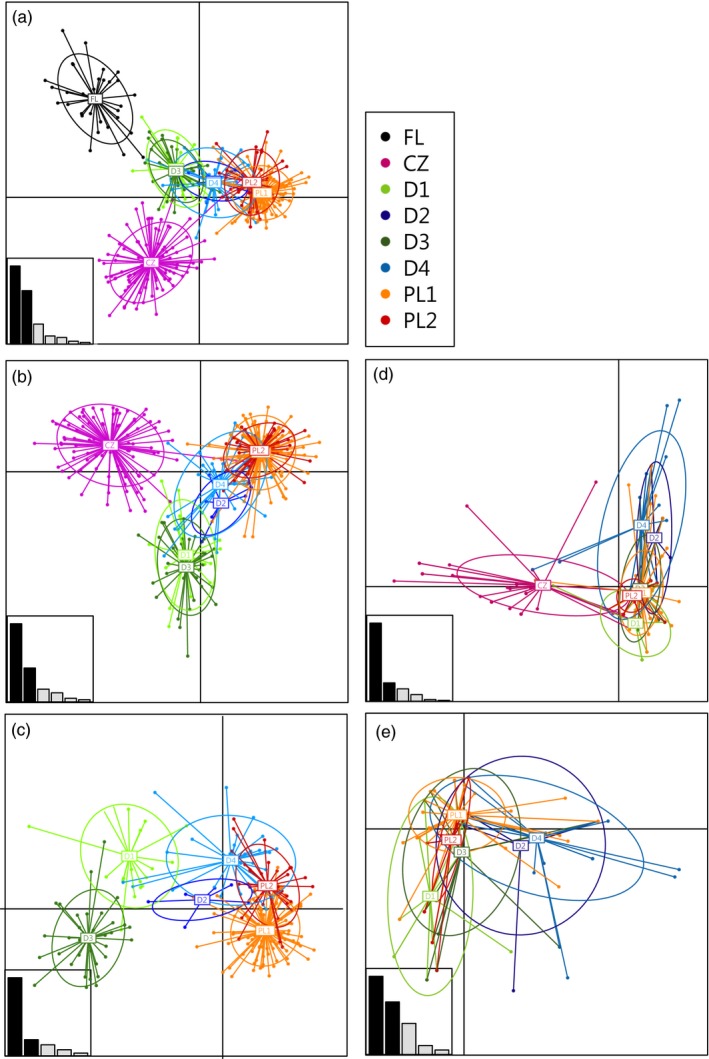
Scatterplot of the genetic differentiation across individuals and sampling sites clusters resulting from a discriminant analysis of principal components (DAPC) for the genetic structure of raccoon individuals based on nine microsatellites (a–c) and MHC‐DRB locus (d–e). Individuals are presented as separate dots with colours denoting sampling locations and inclusion of 95% inertia ellipses. Abbreviations correspond to the sampling locations presented in Figure 1. The inset shows the discriminant analysis (DA) eigenvalues

The discriminant analysis of principal components (DAPC) based on nine microsatellite loci separated native populations from Florida as well as the European CZ site along the first (horizontal) axis. The differentiation between the remaining sites in the invasive range (D1–D4 and PL1–PL2) was visibly weaker and distributed along the second (vertical) axis (Figure 3a). When the native population was excluded, the differentiation of the CZ site was clear and the differentiation between PL and D localities was more pronounced (Figure 3b). After exclusion of CZ, the D sites from the invasion core differentiated along the first axis, while PL sites from invasion front differentiated along the second axis (Figure 3c). The DAPC, on the same hierarchical levels (excluding native population), was performed for the MHC‐DRB locus. The differentiation of CZ from other sites was clearly visible along the horizontal axis (Figure 3d), but no differentiation was visible between the remaining populations. When analysing German and Polish sites only, we failed to find any visible structure (Figure 3e); this was in contrast to microsatellites where the differentiation between populations was visible at the same hierarchical level (Figure 3c).

## 4 DISCUSSION

### 4.1 MHC‐DRB diversity

Newly established invasive populations often exhibit lower levels of genetic diversity in comparison with native populations resulting from the loss of alleles during introduction events (Blackburn et al., 2015; Hagenblad et al., 2015; Tsutsui et al., 2000). Here, we found that indeed, the number of MHC‐DRB alleles is approximately one‐third lower in the invasive range, despite higher sample size compared to the native range (317 individuals from invasive vs. 21 individuals from native range). This result may be due to individuals from the native range comprising different mainland and island regions among which three genetic clusters with limited gene flow were identified (Trujillo & Hoffman, 2017). On the other hand, balancing selection, which is known to maintain MHC polymorphism, promotes retention of the same MHC alleles at the trans‐population and trans‐species levels (Takahata, 1990). Thus, the sampling scheme should not impact our findings. Despite the obvious reduction in allele number in the invasive range, we did not observe a reduction in the number of functional immune variants in the invasive range. The number of supertypes in our invasive raccoon populations ranged from five to seven. Nevertheless, out of ten supertypes that resulted from clustering all available MHC‐DRB alleles (detected in this study and drawn from GenBank), the same nine supertypes were present in the native region we sampled and in the European invasive region. Some degree of differentiation in supertypes present in different invasive sampling sites may be due to contrasting pathogen pressures in these sites, but may also reflect interpopulation differentiation due to demographic processes. Still, taking into account high levels of migration and the subsequent spread of the invasive raccoon population, we can expect the presence of almost all functional variants in the invasive range in a short time, which may contribute to the success in new environment. The maintenance of diverse immune functional variants is also reflected in high divergence found among alleles at the individual level and, more importantly, high number of supertypes possessed by individuals in the invasive populations. This would suggest that the loss of genetic variants during the invasion to a new environment did not necessarily lead to the loss of functional diversity. In fact, the retention of MHC diversity after a bottleneck event, in terms of nucleotide diversity and divergent allele sets, has been already been observed in other studies of MHC genes in natural populations (Marmesat, Schmidt, Saveljev, Seryodkin, & Godoy, 2017; Oosterhout et al., 2006; Vlček et al., 2016) and was interpreted as the footprint of balancing selection in the form of divergent allele advantage.

To infer if individual supertype diversity is the effect of selection that acts on maintaining the highest number of divergent immune variants or the random effect that took place during the establishing of invasive population, we compared observed mean individual supertype number to the one represented by genotypes simulated from alleles observed in the populations. Taking into account that drift should first reduce the number of alleles, but not supertypes, as every supertype is represented by at least several alleles, it is possible that maintaining supertypes while alleles are lost is only a random effect. In the case of duplicate MHC loci, when alleles cannot be assigned to particular loci, there is no straightforward method of distinguishing between the effect of drift and balancing selection. Without gene conversion that homogenizes alleles between paralogous loci, genetic drift would result in the retention of divergent alleles (and higher number of MHC supertypes as a consequence) and at least one representative of each divergent lineage would be kept in each locus. In such a case, it is not parsimonious to invoke balancing selection.

In the invasive population extending north‐east from the invasion source in Germany to western Poland (sites PL and D), the simulated genotypes represented lower mean individual supertype number when random segregation of alleles was assumed, but higher supertype number with the assumption of allele association, according to the pattern present in the studied populations. This finding suggests that the maintenance of divergent alleles and high number of supertypes is a result of linkage disequilibrium between alleles. All pairs of linked alleles found in this study are placed far apart on allele network (Figure 2) and represent two different supertypes, which suggest the role of disassortative mating in creating the associations between alleles. The role of MHC supertype dissimilarity in disassortative mating was proven for grey mouse lemur (Schwensow, Eberle, & Sommer, 2008). However, as we are not able to assign alleles into loci, we also cannot rule out the linkage resulting from the close proximity of alleles on the chromosome. This result is in line with the pattern found in Czech population, where no difference was observed between observed and simulated data, and only one pair of linked alleles was found.

The process that can explain the emergence of allele associations is balancing selection operating between rather than within loci that will reinforce linkage disequilibrium through epistasis. Such a process would allow the extension of the target of selection to multilocus MHC haplotypes (Innan & Kondrashov, 2010; Wakeland et al., 1990). This mechanism was suggested for interactions between HLA loci and pathogens or disease (Lincoln et al., 2009; Penman, Ashby, Buckee, & Gupta, 2013). In our study, three out of five linked allele pairs consisted of alleles that were previously shown to give resistance to rabies (Srithayakumar, Castillo, Rosatte, & Kyle, 2011). All those three alleles (*Prlo*‐DRB*4, *Prlo*‐DRB*16 and *Prlo*‐DRB*102) were present and frequent in Polish and German populations, while *Prlo*‐DRB*4 and *Prlo*‐DRB*16 were found in Czech population (Table S3). Our study lacks data on rabies prevalence, but according to the literature, in the invasive raccoon range rabies was observed rarely and outside the region studied here (Beltrán‐Beck, García, & Gortázar, 2012). So, high frequencies of those three alleles may be the result of strong directional selective pressure that took place before raccoon were introduced to Europe. Moreover, this may provide protection for the raccoons from rabies in the invasive range and, as a consequence, we observe the effect of linkage of individual supertypes.

Interestingly, we found strong support for divergent variant advantage acting in the native range. The random genotypes simulated from native population represent, on average, significantly lower numbers of supertypes in comparison with sampled population, implying that the selection process in response to pathogen pressure favours divergent variant combinations in the native range. This result is in line with the prediction of Lee (2016) and observations of Posavi et al. (2014) who found that high level of genetic diversity in native populations maintained by balancing selection enables adaptation to the novel environment in the invasive range. This is why genetic diversity is one of the most important conditions determining “invasiveness” of a population. Although our results from the invasive range do not support clearly the presence of divergent variant advantage, there is evidence that this process might still be playing an important role in invasive raccoon populations. A study of raccoons' mating preferences, performed in one of the introduced populations, demonstrated the role of disassortative mating in individual MHC class I diversity. Additionally, high MHC I diversity was related to increased chances of fathering offspring (Santos, Mezger, Kolar, Michler, & Sommer, 2018; Santos et al., 2017). It is well established that MHC genetic diversity is maintained by balancing selection (Hedrick, 2002), and we argue that in the case of raccoons, maintaining diverse functional immune variants at the individual level in the native range is an important feature that facilitates raccoon success in the invasive range.

Our study covered a wide range of the raccoon distribution throughout Europe, both from the invasion core and from the invasion front. Given such a wide sampling scheme, one would expect to find reduced genetic diversity along the invasion wave (Corre & Kremer, 1998; Estoup & Guillemaud, 2010; Lawson Handley et al., 2011). Although such a pattern seems to hold for neutral mitochondrial genetic diversity (Biedrzycka et al., 2014; Frantz et al., 2013), it was not confirmed by microsatellites (Biedrzycka et al., 2014) or MHC diversity, markers that are generally more diverse than mitochondrial DNA. Moreover, when analysing MHC‐DRB diversity in terms of number of alleles per site, allelic richness or mean number of alleles per individual, there were no pronounced differences between sampling sites. The lack of a diversity gradient, along with the lack of private alleles, suggests mixing of individuals from different introduced areas in the new colonized range. On the other hand, the allele frequencies in D1, the location of the first recorded raccoon introduction (Jernelöv, 2017), was the only site characterized by evenly distributed allele frequencies. Such a pattern can be explained by some form of balancing selection (Mukherjee, Sarkar‐Roy, Wagener, & Majumder, 2009; Takahata, 1990) that plays an important role in shaping MHC diversity at an invasion core (White & Perkins, 2012), while the diversity of newly established populations at the invasion edges may be shaped more by genetic drift.

### 4.2 MHC‐DRB structure

We assessed the genetic structure of invasive racoon populations at the MHC‐DRB locus and selectively neutral microsatellite loci to evaluate the relative power of demographic events and selection in shaping the diversity of this functional locus. Furthermore, we sought to determine how the immune genetic diversity that enables adaptation to novel environments is distributed in invasive populations. According to the results of the Mantel test and partial Mantel test, we were not able to draw a clear interpretation if demographic or selective processes shaped interpopulation MHC diversity. We found a significant correlation between microsatellite diversity and geographic distance, while the same correlation was not significant with regard to MHC‐DRB diversity. Although this could be suggestive of different processes shaping the diversity of neutral and adaptive markers, the difference in statistical significance was not pronounced and could reflect higher discriminatory power of nine microsatellite loci in comparison with the one MHC‐DRB locus in finding population structure. Furthermore, while controlling MHC–geographic distance relationship for neutral diversity, there was no association, suggesting that population structure at MHC‐DRB locus likely reflects the processes of raccoon population dispersal.

Discriminant function of principal components, the method that does not assume Hardy–Weinberg or linkage equilibrium, gave more informative results with regard to interpopulation structure. By using principal component analysis (PCA) to reduce the number of variables, it does not rely directly on allele numbers and frequencies, which makes it more appropriate to analyse and compare two different types of genetic markers. Regardless of the marker type, the raccoons from CZ were clearly distinct from the other sites as shown by DAPC. This is in line with the findings presented in Biedrzycka et al. (2014) and supports a separate origin of raccoons at this site. The lack of genetic linkage between Prlo‐DRB*04 and Prlo‐DRB*14 alleles at CZ provides further evidence that this site has a unique origin, although we cannot exclude the scenario of different selective pressure acting in this population. The separate ancestry of Czech population may be further supported by the dates of first observation of raccoon in Czechia in the 1950s (Červeny, Andĕra, Koubek, Homolka, & Toman, 2001), while the expansion of raccoons from Germany did not start before the 1960s (Lutz, 1984). Considering the remaining invasive populations, the structure is clearly visible for microsatellite data, but absent for the MHC‐DRB locus. If the selection pressure is uniform across different sampling sites, lower differentiation should be observed at functional loci because of balancing selection maintaining functional alleles across different sites (Oosterhout et al., 2006), which may be the case here. In a study of fluctuating population sizes of water voles (Bryja, Charbonnel, Berthier, Galan, & Cosson, 2007), balancing selection acted on the MHC locus during the high‐abundance year. In our study, we also found lower differentiation displayed by the immune‐related locus, at high migration rates during intensive population spread (Jernelöv, 2017). This pattern suggests uniform selection pressures exerted by pathogens in Polish and German raccoon populations. Importantly, the invasive raccoon populations are characterized by high population numbers that would act to increase the efficiency of balancing selection.

### 4.3 Management implications

Our data suggest that raccoon MHC genes are highly diverse and may represent multiple invasions. Additionally, known and unknown introduction events (including escapes from zoos; Fischer et al., 2017) and hybridization between raccoons introduced to Czechia and Germany may further increase the genetic variation of MHC genes that promote higher raccoon adaptation. Poland was already colonized from both directions: south (Czechia) and west (Germany) with the contact zone in the western central region (Biedrzycka et al., 2014). We should expect similar patterns in western Europe between raccoons introduced to Spain and Germany, as those populations also belong to different genetic clusters (Fischer et al., 2017).

The increase in diversity after secondary contact may have substantial management implications as raccoons may be able to rapidly adapt to pathogens in newly colonized ranges. Additionally, native and introduced populations exhibited similar patterns of historical selection at functional sites of the MHC‐DRB locus, which may suggest that there are similar pathogen pressures in both regions. Indeed, the important pathogens that have affected racoon populations are rabies, canine distemper and *Baylisascaris procyonis*, which occur in North America and Europe (e.g. Blizzard, Yabsley, Beck, & Harsch, 2010; Martinez‐Gutierrez & Ruiz‐Saenz, 2016; Popiołek et al., 2011; Reynolds, Hirsch, Gehrt, & Craft, n). As these pathogens were historically present in native populations, there seem to be some adaptations in the hosts that provide resistance or reduce the effects of these severe infections. Indeed, the alleles that were the most frequent in European population, *Prlo*‐DRB*4, *Prlo*‐DRB*16 and *Prlo*‐DRB*102, were found to give resistance to rabies (Srithayakumar et al., 2011). We can expect that the enhanced survival of carriers of these alleles may further boost invasion success.

A successful programme for reducing raccoon invasive populations may be difficult in Europe. A management strategy should rely on the knowledge of neutral and adaptive genetic diversity and structure of the species to recognize its evolutionary potential. Most importantly, the management action for raccoons in Europe should act to reduce cryptic introductions (especially from private breeding and zoos) as such events potentially add more genetic variation and can introduce novel adaptive alleles into invasive populations. Moreover, wildlife managers should pay attention to reducing the possibility of mixing raccoons belonging to different genetic clusters. To do so, the efforts should be concentrated on preventing the further spread of raccoons forming populations at the invasion front. Furthermore, to reduce MHC diversity, a management plan should focus on reducing raccoons at source habitats where the racoon numbers and genetic diversity are the highest. One important question that still needs to be answered is: What is the role that urban populations play in adding functional genetic diversity and zoonotic disease risk to native wildlife and humans?

## 5 CONCLUSIONS

Our research showed that functional genetic diversity is maintained in invasive raccoon population, despite a strong reduction of MHC‐DRB allele number. We found that invasive raccoons have high number of functionally important MHC supertypes and individual allele divergence compared to the native populations. Additionally, low population structuring displayed at the MHC‐DRB locus than in microsatellites implies the role of balancing selection. Many studies have found that despite experiencing genetic bottlenecks, successful invasive populations retain a higher level of genetic diversity than expected, probably due to multiple introduction events (Uller & Leimu, 2011), but research concerning functional genetic diversity of successful invaders is lacking (but see Monzón‐Argüello et al., 2014; Wellband et al., 2018).

Here, we showed that immune genetic diversity might be retained despite a strong reduction in the number of MHC alleles. Because MHC loci capture an important fraction of the genetic variation underpinning resistance to pathogens, we predict that raccoon invasion in Europe will proceed based on observed levels of functional genetic diversity.

## CONFLICT OF INTEREST

None declared.

## Data Availability

The DNA sequences obtained in this study are deposited in GenBank under accession numbers MK028988–MK028995. The files containing the results of MHC‐DRB locus genotyping and R scripts used for simulation of mean individual supertype number have been deposited in the Dryad Digital Repository under the following DOI: https://doi.org/10.5061/dryad.rr4xgxd55 (Biedrzycka, Konopiński, Hoffman, Trujillo, & Zalewski, 2019).
